# Performance of Total Blood Volume Algorithms in Obesity and Severe Obesity

**DOI:** 10.1002/jca.70038

**Published:** 2025-06-13

**Authors:** Caitlin Raymond, Ninet Sinaii, Kamille West‐Mitchell

**Affiliations:** ^1^ Department of Transfusion Medicine National Institutes of Health Clinical Center Bethesda Maryland USA; ^2^ Biostatistics and Clinical Epidemiology Service National Institutes of Health Clinical Center Bethesda Maryland USA

**Keywords:** algorithms, apheresis, obesity, physiology, severe obesity, total blood volume

## Abstract

Amid the ongoing obesity epidemic, the estimation of total blood volume (TBV) in obese people remains hotly contested without common agreement among apheresis practitioners. We compare the results of estimated TBV from different formulas across a spectrum of obese BMIs from a cohort of 155 individual patients. We also plot the difference between the resulting TBVs, assigning a threshold of functional significance of 500 mL, at which the choice of formula might make an impact on patient care. We compare the mean TBV estimated in non‐severe versus severe obesity for all the above algorithms and determine the impact of algorithm choice on various apheresis procedures. The choice of algorithm has a significant impact on apheresis procedures; for example, we found differences of up to ~3–4 plasma units for a therapeutic plasma exchange depending on the choice of algorithm applied. We additionally find that algorithm performance varies widely in both men and women, particularly in morbid obesity, and often produces values that fall outside an empirically chosen expected range. We do not find a clinically significant difference in mean estimated TBV between non‐severe and severe obesity in any algorithm tested, suggesting that physiological changes in obesity may fail to be captured by these algorithms. We hope these results are useful to other apheresis practitioners and help them make an informed choice of algorithm to estimate TBV in their obese patients. However, our current algorithms for estimating TBV may be flawed, and the field may wish to move toward measurement of TBV using recently available commercial options.

## 1 Introduction

Over the past 40 years obesity has been on the rise in the United States, with the average body mass index (BMI) increasing from 25.7 in 1971 to 30.0 in 2020 [1, 2, 3]. Obesity is defined as a BMI ≥ 30.0, with severe obesity occurring with a BMI between 35.0 and 39.9, and morbid obesity occurring with a BMI ≥ 40.0 [1, 2]. While much research has gone into the health problems associated with obesity, there is relatively little known about the physiologic changes produced with this condition [4]. In apheresis, accurate determination of the total blood volume (TBV) is critical to achieve the therapeutic effect or collect the desired cells of interest, while avoiding adverse effects. However, there is no widely accepted method of estimating TBV in obese patients. While it seems intuitive that a larger body habitus would result in a larger TBV, this may not accurately reflect the physiology of obesity. Studies have shown that lean body mass is the most accurate predictor of TBV [5, 6, 7, 8], and that this may be decreased in certain types of obesity, although not all [9]. Similarly, research has demonstrated that adipose tissue contains fewer capillary beds [10], and may have reduced blood flow in obesity [11]; thus, the relationship between weight and blood volume is most likely not linear and remains poorly understood.

Several methods exist to measure TBV, such as nuclear medicine blood volume assessment (NM‐BVA) [12, 13] and carbon monoxide (CO) re‐breathing [14, 15]. Historically, these methods have been expensive, cumbersome, and time‐consuming, and did not lend themselves to routine patient care. In lieu of direct measurement, several equations were developed to estimate TBV for clinical purposes. Nadler's formula was developed in 1962 based on NM‐BVA measurement of a cohort of healthy, normal‐weight volunteers, and is widely considered the most accurate method of estimating TBV [16, 17, 18]. However, there are no modifications to the formula specific to obesity, and it may underestimate TBV in modern populations by up to 15% [19]. Gilcher's rule of fives involves multiplying the person's weight in kg by a population average in mL/kg to produce an estimated TBV [16]. Gilcher's rule is commonly used as a rough estimate of TBV, and there are population averages available for normal weight men and women as well as obese men and women [20]. Finally, the Lemmens–Bernstein formula will also produce an estimated TBV and was specifically designed for use in obese patients. Developed in 2006, the Lemmens–Bernstein formula was originally intended for use with pharmaceutical dosing but has been used for apheresis calculations in recent years [17, 21].

With all this variety of formulas to estimate TBV in obese people comes a variety of practices. A recent focus group found that there was no common agreement for estimating TBV in obese persons among apheresis practitioners; all the above equations were used by different institutions, and in some cases different algorithms were used in different patients in the same institution [22]. Moreover, these equations were variously used with actual body weight (ABW), ideal body weight (IBW), or by modifying IBW to produce an adjusted IBW (AIBW) [23, 24]. In a related survey, only 20% of the responding institutions had a policy for estimating total plasma volume (TPV) in obese persons, which requires estimation of TBV [24].

To help shed light on best practices, we perform a theoretical exercise to compare the parameters of common apheresis procedures based on estimated TBV as calculated by existing formulas. We used data from a hypothetical patient with fixed characteristics to compare TBV estimates generated by different formulas and the potential impact of the different estimates on apheresis procedures. Next, we compared the estimated TBV produced by these formulas for a cohort of 155 obese patients who underwent apheresis in our institution. To help determine the BMI at which the choice of algorithm might make an impact on patient care, we plotted the difference in TBV across our patient‐derived dataset and compared it to an empirically defined threshold of functional significance. In addition, we analyzed the mean TBV in non‐severe versus severe obesity to test whether these algorithms produce clinically significant differences in TBV as obesity progresses. These results should help guide other practitioners in making the best choices for their patients.

## 2 Ethics Approval Statement

This study was determined by the NIH institutional review board (IRB) to constitute exempt human subjects research per 45CFR 46.104(d) (4)(ii) (submission # IRB002364).

## 3 Materials and Methods

### 3.1 Comparing Estimated TBV Formulas Using a Standardized Hypothetical Patient

To facilitate a direct comparison of TBV estimates across multiple formulas, we applied each equation to a standardized hypothetical patient (height: 173 cm, weight: 120 kg, BMI: 40, hematocrit: 40%). This approach allows for consistent input variables and enables evaluation of the differences attributable solely to the formulas themselves, independent of patient‐specific variability. For this hypothetical patient, we calculated TBV, red cell volume (RCV), total plasma volume (TPV), IBW, and AIBW. We used Nadler's formula, the Lemmens–Bernstein formula, and Gilcher's rule of fives (Table 1). For Nadler's formula the AIBW was used. Gilcher's rule was calculated using AIBW and a constant of 60 mL/kg for obese men and a constant of 55 mL/kg for obese women.

**TABLE 1:** Summary of equations used in this study.

Formula	Equation
BMI	Weight in kg/(height in m^2^)
Devine's formula	Male: 50 + (0.91 × [height in cm − 152.4])
	Female: 45.5 + (0.91 × [height in cm − 152.4])
Adjusted IBW	IBW + (0.25 × (ABW − IBW))
Nadler's formula	Male: 604.1 + (0.0003668 × (height in cm^3^)) + (32 × weight in kg)
	Female: 183.3 + (0.000356 × (height in cm^3^)) + (33 × weight in kg)
Lemmens–Bernstein formula	(70 mL/kg × ABW in kg)/√(BMI/22)
Gilcher's rule	Male: Weight kg × 60 mL/kg
	Female: Weight kg × 55 mL/kg

Abbreviations: ABW, absolute body weight; AIBW, adjusted ideal body weight; BMI, body mass index; IBW, ideal body weight.

We then sought to determine the impact of the differences in TBV estimates on apheresis procedures. Using the standardized hypothetical patient data described above, we determined the number of blood products that would be required for replacement fluid during red blood cell exchange (RCE) and therapeutic plasma exchange (TPE) based on the different TBV estimates. For these calculations, we assumed an erythrocyte volume of 200 mL in a standard unit of RBC, as the practice at our institution is to calculate RBC exchanges based on erythrocyte volume. We additionally assumed a plasma unit volume of 300 mL. For RCE, we assumed the desired fraction of red cells remaining (FCR) was 30%. For TPE, we assumed a 1.0 volume plasma exchange procedure.

We also attempted to assess the impact of these various algorithms on cell collections, which typically involve processing up to five blood volumes. Using the same hypothetical patient, we calculated 5x TBV using each formula and determined the differences in TBV to be processed, as well as the estimated time to complete the procedure assuming a flow rate of 80 mL/min.

Decimals ≥ 0.5 were rounded up, and decimals < 0.5 were rounded down. All formulas and calculations used in this manuscript can be found in Table 1.

### 3.2 Comparing Estimated TBV Formulas in a Patient Cohort

Next, we sought to compare the performance of these various algorithms in a real‐world dataset derived from obese patients who underwent apheresis at our institution. Weight, height, and sex data were collected from adult patients who underwent apheresis for allogeneic or autologous cell collection in our institution between 2010 and 2021. We used Nadler's formula, the Lemmens–Bernstein formula, and Gilcher's rule of fives (Table 1). For Nadler's formula the AIBW was used, and Gilcher's rule was used with both ABW and AIBW. The results across a spectrum of obese BMIs were plotted separately for women and men, given the sex‐specific constants present in some equations.

BMI for all patients was calculated, then data points were filtered for BMIs greater than 30.0. All data points from men and women were separated. A total of 58 unique data points were collected from women, and a total of 97 unique data points were collected from men. IBW was calculated using Devine's formula. AIBW was calculated as previously described [23]. Then, calculations to determine estimated TBV were performed using the following: height in cm where applicable, with either ABW in kg or AIBW [23]. All formulas and calculations used in this manuscript can be found in Table 1. Line plots were created in Microsoft Excel using standard functions.

### 3.3 Definition of Expected Range

While the literature agrees that TBV is likely higher in obese individuals, there is also consensus that the relationship between TBV and body habitus is not linear. Thus, it is unlikely that TBV would reach extremes of over 7 L predicted by various algorithms in higher BMIs. To compare the performance of these algorithms, we chose empiric expected ranges for obese men and women. These empiric ranges were selected based on prior literature showing measured TBV ranges of 4500–6500 mL in normal weight adult men [25]. For this study, we chose a more generous 3 L range for obese men of 4000–7000 mL to broadly capture acceptable results. For women, we adjusted this range down to 3500–6500 mL based on previous findings that women have lower measured TBV values [18, 19, 26]. We hypothesize that TBV should fall within these expected ranges if the algorithm performs well.

### 3.4 Definition of Functional Significance

For this study, a difference in estimated TBV of ≥ 500mL was defined as the functionally significant threshold. Assuming a hematocrit of 40%, a difference of ~500 mL of TBV would indicate ~300 mL of plasma volume and would necessitate the exchange of an additional unit of plasma (~300 mL/unit) in a 1.0 volume plasma exchange procedure. A difference of 500 mL in TBV would also produce differences of 2–3 L in cellular collections when processing 5–6x TBV, making this a broadly applicable functional threshold.

### 3.5 Mean TBV in Non‐Severe and Severe Obesity

The mean estimated TBV was compared in persons with non‐severe obesity (BMI of 30.0–34.9) versus severe obesity (BMI ≥ 35.0). We had relatively few data points for individuals with morbid obesity (BMI ≥ 40.0), these were combined with the severe obesity cohort.

### 3.6 Statistical Analysis

Generalized linear models were used as an extension of multiple two‐tailed, two‐sample *t* tests to compare TBV in non‐severe and severe obesity for each of males and females. Data were assessed for and met distributional and statistical assumptions. Where applicable, data are described or shown using arithmetic mean and standard deviation. Data were analyzed using SAS v9.4 (SAS Institute Inc., Cary, NC).

## 4 Results

### 4.1 Impact of Various Algorithms on Apheresis Procedures in a Standardized Hypothetical Patient

For our hypothetical patient, the results of TBV, RCV, and TBV as calculated by each formula are summarized in Table 2. For RCV, comparing the lowest value estimated (from Gilchers‐55) with the highest value estimated (from Lemmens–Bernstein), we see a difference of 695 mL or ~3–4 RBC units (Table 2). For TPV, comparing the lowest value estimated (from Gilchers‐55) with the highest value estimated (from Lemmens–Bernstein), we see a difference of over 1000 mL or ~3–4 plasma units (Table 2). Thus, the choice of algorithm produces major impacts on the parameters of both RBC and plasma exchange.

**TABLE 2:** Impact of algorithms on red blood cell and plasma exchange.

Formula	TBV (mL)	RCV (mL)	TPV (mL)	RBC (units)	Plasma (units)
Lemmens–Bernstein	6222	2489	3733	15	12
Gilcher's rule AIBW‐60	4894	1957	2936	12	10
Gilcher's rule AIBW‐55	4486	1794	2691	11	9
Nadler's AIBW female	4718	1887	2831	11	9
Nadler's AIBW MALE	5113	2045	3068	12	10

*Note:* A constant height of 173 cm and weight of 120 kg, which produce a BMI of 40, were entered into each of seven algorithms to estimate a total blood volume (TBV). A constant hematocrit of 40% was used to calculate red cell volume (RCV) and total plasma volume (TPV) in each case. The number of red blood cell (RBC) units and plasma units required for an exchange were calculated assuming a fraction of cells remaining (FCR) of 30%, an RBC volume of 200 mL/unit, a 1.0 volume plasma exchange procedure, and a plasma volume of 300 mL/unit.

Abbreviations: BMI, body mass index; Gilcher's rule AIBW‐55: Gilcher's rule using AIBW and a constant of 55 mL/kg, as recommended for obese women; Gilcher's rule AIBW‐60, Gilcher's rule using AIBW and a constant of 60 mL/kg, as recommended for obese men; Lemmens–Bernstein, the Lemmens–Bernstein algorithm; Nadler's AIBW female, Nadler's equation for female patients using AIBW; Nadler's AIBW male, Nadler's equation for male patients using AIBW.

For our hypothetical cell collection, the blood volumes to be processed predicted by Lemmens–Bernstein and Gilcher's rule‐55 were the most disparate, with the 5x TBV processing volume differing by 8682 mL (Table 3). This would result in an approximate 1.8 h difference in procedure length, assuming a flow rate of 80 mL/min (Table 3). Such a difference could lead to a large impact on the number of cells collected in a procedure, potentially necessitating a second collection with all the inherent risks.

**TABLE 3:** Impact of algorithms on cell collections.

Formula	TBV (mL)	5x TBV collection (mL)	Hours
Lemmens–Bernstein	6222	31 111	6.5
Gilcher's rule AIBW‐60	4894	24 468	5.1
Gilcher's rule AIBW‐55	4486	22 429	4.7
Nadler's AIBW female	4718	23 590	4.9
Nadler's AIBW male	5113	25 566	5.3

*Note:* A constant height of 173 cm and weight of 120 kg, which produce a BMI of 40, were entered into each of seven algorithms to estimate a total blood volume (TBV). A 5x TBV cell collection was calculated, indicating the total volume to be processed. The length of the procedure in hours was estimated assuming a flow rate of 80 mL/min.

Abbreviations: BMI, body mass index; Gilcher's rule AIBW‐55, Gilcher's rule using AIBW and a constant of 55 mL/kg, as recommended for obese women; Gilcher's rule AIBW‐60, Gilcher's rule using AIBW and a constant of 60 mL/kg, as recommended for obese men; Lemmens–Bernstein, the Lemmens–Bernstein algorithm; Nadler's AIBW female, Nadler's equation for female patients using AIBW; Nadler's AIBW male, Nadler's equation for male patients using AIBW.

### 4.2 Comparing Estimated TBV Formulas in Obese Patients

**FIGURE 1:**
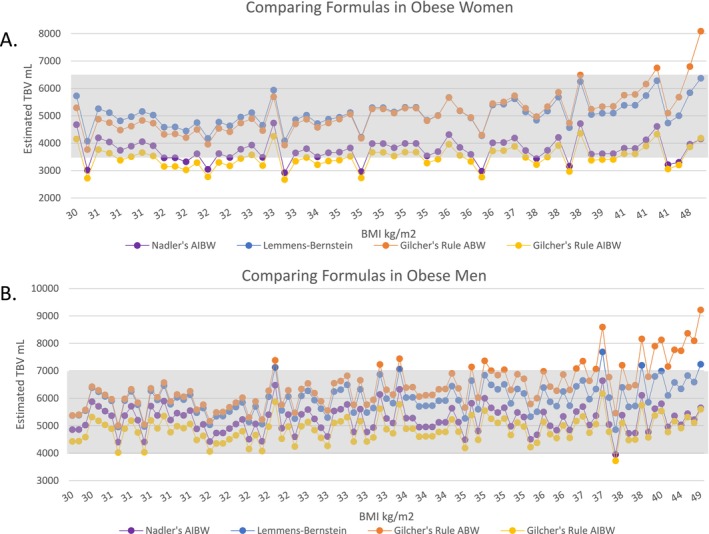
Comparing the performance of TBV estimation formulas. (A) Estimated TBV from four algorithms is plotted across a range of BMIs for obese women. An expected range of 3500–6500 mL is shaded in gray. (B) Estimated TBV from four algorithms is plotted across a range of BMIs for obese men. An expected range of 4000–7000 mL is shaded in gray. The algorithms are Nadler's formula with AIBW (purple line), Lemmens–Bernstein formula (blue line), and Gilcher's rule with ABW (orange line) or AIBW (yellow line). ABW, actual body weight; AIBW, adjusted ideal body weight; BMI, body mass index; TBV, total blood volume.

In women, both Nadler's formula using the AIBW and Gilcher's rule using the AIBW produced TBV values at the lower spectrum of expected TBV (3500–6500 mL, shaded in gray, Figure 1A), in many cases notably undershooting the lower bound at lower BMIs. Both formulas produce similar results to each other across the entire spectrum of BMI plotted, even in the morbidly obese range. Nadler's formula produced a range of 2922–4736 mL with a 50th percentile of 3759 mL, and Gilcher's rule with AIBW produced a range of 2668–4357 mL with a 50th percentile of 3479 mL.

In contrast, the Lemmens–Bernstein formula and Gilcher's rule with ABW produced values at the upper range of expected TBV, with ranges of 4072–6372 and 3768–8091 mL, respectively. Lemmens–Bernstein produced a 50th percentile of 5070 mL, and Gilcher's rule with ABW produced a 50th percentile of 4989 mL. In morbid obesity, there was overshooting of the upper bound of the expected range with the Lemmens–Bernstein formula, and more prominent overshooting with Gilcher's Rule with ABW. Notably, at BMIs ≥ 40.0, there appears to be a substantial spread in the estimated TBV calculated with all four formulas plotted, ranging in one case from 4 to 8 L (Figure 1A). Comparing the performance of all four formulas, the Lemmens–Bernstein formula produces the most values in the expected range; thus, it was considered the best performing.

In men, Nadler's formula with AIBW and Gilcher's rule with AIBW both produce values within the expected range (4000–7000 mL, shaded in gray, Figure 1B). The ranges are 3951–6637 and 3718–6166 mL with 50th percentiles of 5223 and 4867 mL, respectively. The Lemmens–Bernstein formula and Gilcher's rule with ABW produce values at the upper limit of, or even overshooting, the expected range. We found ranges of 4850–7684 mL for Lemmens–Bernstein and 5004–9216 mL for Gilcher's rule with ABW, with 50th percentiles of 5948 and 6306 mL, respectively. Overshooting is most pronounced in severe and morbid obesity (BMI ≥ 35.0), and again we see a wide spread in the TBV estimated by all four methods in this obesity class, with one instance of a range of 6–9 L (Figure 1B). Comparing the performance of all four formulas plotted, Nadler's formula with AIBW and Gilcher's rule with AIBW produce the most values in the expected range, without notable overshooting or undershooting, and thus were considered the best performing.

### 4.3 Functional Significance of Estimated TBV in Obese Persons

**FIGURE 2:**
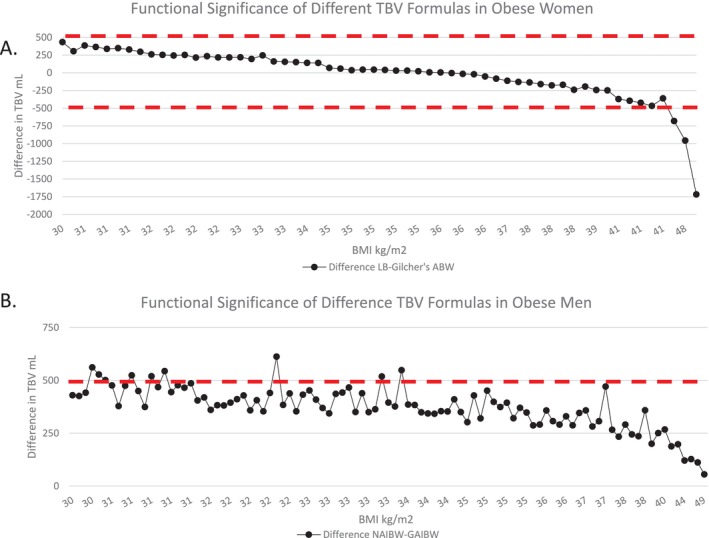
Functional significance of different TBV formulas. (A) The difference between Lemmens–Bernstein formula minus Gilcher's rule with ABW is plotted across a range of BMIs for obese women. (B) The difference between Nadler's formula with AIBW (NAIBW) minus Gilcher's rule with AIBW is plotted across a range of BMIs for obese men. Red dashed lines indicate our threshold of significance (500 mL). ABW, actual body weight; BMI, body mass index; GAIBW, Gilcher's rule with adjusted ideal body weight; LB, Lemmens–Bernstein; NAIBW, Nadler's formula with adjusted ideal body weight; TBV, total blood volume.

That different formulas produce different estimated TBVs is not unexpected, but we sought to address the question of when this might make a functional impact on patient care. To determine this, we plotted the difference between the best performing formulas in Figure 1 across the spectrum of BMI in our dataset and set a threshold of functional significance of 500 mL (defined above). In Figure 2A, we plot the difference between the Lemmens–Bernstein formula and Gilcher's formula with ABW in obese women, as these two formulas produced values that best fit our expected range in Figure 1A. We find that at lower BMIs, the Lemmens–Bernstein formula produces slightly higher TBV estimates that do not reach our threshold of functional significance. In contrast, at higher BMIs, the Lemmens–Bernstein formula produces lower TBV estimates, well below our threshold for functional significance.

To determine the functional significance of different TBV estimations in men, we plotted the difference of Nadler's formula with AIBW and Gilcher's rule with AIBW (Figure 2B), since these two formulas best fit our expected range in Figure 1B. Generally, our threshold of functional significance is not reached, with some exceptions at lower BMIs. As the BMI increases, the differences in TBV decrease, and in the morbidly obese range, the two formulas produce TBV estimates that are arguably interchangeable.

### 4.4 Mean TBV in Non‐Severe and Severe Obesity

In the above figures, we identify the Lemmens–Bernstein formula in obese women and either Gilcher's rule with AIBW or Nadler's formula with AIBW in obese men as best performing, meaning they produced the most values in our expected range. While these formulas are commonly used, their performance in different classes of obesity is untested. To help shed light on these algorithms, we determined the mean estimated TBV in non‐severely obese (BMI 30.0–34.9) and severely obese (BMI ≥ 35.0) men and women. In doing so, we test whether these algorithms produce significantly different results as obesity progresses in severity.

**FIGURE 3:**
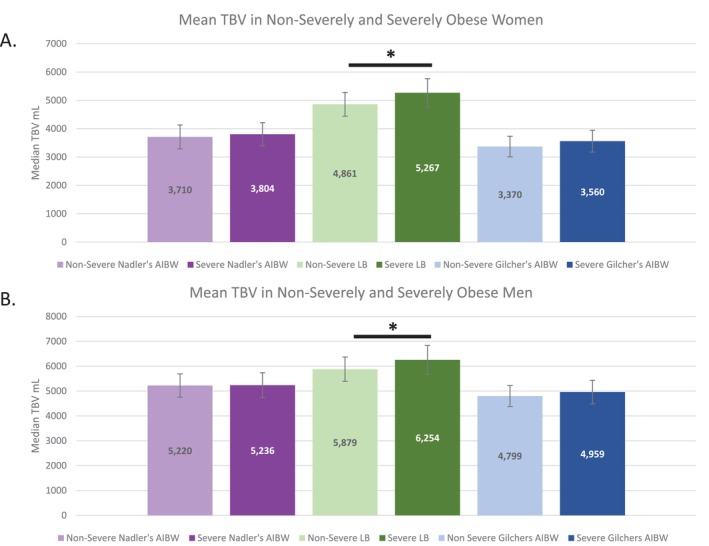
Comparison of TBV non‐severe and severe obesity. (A) The mean TBV in non‐severely (BMI of 30.0–34.9) or severely (BMI ≥ 35.0) obese women for three different formulas is presented. *p = 0.003. (B) The mean TBV in non‐severely (BMI of 30.0–34.9) or severely (BMI ≥ 35.0) obese men for three different formulas is presented. *p < 0.001. AIBW, adjusted ideal body weight; LB, Lemmens–Bernstein formula; TBV, total blood volume.

In both men and women, we found no statistically significant difference in TBV between non‐severe and severe obesity when TBV was calculated with either Nadler's formula using AIBW or Gilcher's rule with AIBW (Figure 3A,B). However, TBV calculated with the Lemmens–Bernstein formula showed statistical differences between non‐severe and severe obesity in both men (5879 vs. 6254 mL, *p* < 0.001) and women (4861 vs. 5267 mL, *p* = 0.003). While the Lemmens–Bernstein formula did produce an increased TBV in severe obesity in both datasets, we note that the difference did not meet our threshold of functional significance (≥ 500 mL).

## 5 Discussion

The question of how to appropriately estimate TBV for obese patients has puzzled apheresis practitioners for some time. Currently, there are a range of formulas to estimate TBV that can further be used with a number of non‐adjusted or adjusted body weights, making the selection of the most appropriate method overwhelming. In this study, we perform a theoretical exercise with a standardized dataset to estimate the clinical impact different TBV algorithms might have on a variety of apheresis procedures. We find that different algorithms can produce widely disparate apheresis parameters, with differences of ~3–4 units of RBC for a red cell exchange, ~3–4 plasma units for a TPE, or ~1.8 h of processing time for a cell collection. We then compared the performance of several algorithms commonly used to estimate TBV in a real‐world dataset derived from obese patients who had undergone apheresis at our institution. Again, we found widely disparate results. We next attempt to determine the functional impact of using different TBV algorithms by determining the BMI at which differences in estimated TBV reach clinical significance (> 500 mL). Finally, we also compare the difference in mean estimated TBV in non‐severe and severe obesity.

We found that different formulas perform best in obese women versus obese men, likely due to the sex‐specific modifiers present in these formulas. In obese women, we found that current formulas demonstrate either undershooting of an expected range at lower BMIs or overshooting of that expected range in higher BMIs, with the Lemmens–Bernstein formula producing the best fitting results. In contrast, we found that Nadler's formula with AIBW or Gilcher's rule with AIBW performed well in obese men but still had isolated instances of undershooting or overshooting the expected range. Thus, none of the formulas we tested truly performed within the expected range.

Apart from the Lemmens–Bernstein formula, none of the formulas tested produced a statistically different TBV in severe obesity compared to non‐severe obesity in either men or women. Moreover, the difference in mean TBV calculated with the Lemmens–Bernstein formula did not reach our threshold of clinical significance (> 500 mL). While this analysis was performed to uncover a significant difference in TBV as obesity progresses in severity, this hypothesis involves making two assumptions which may not be correct. First, there is limited evidence on physiologic changes in obesity, and what evidence we do have suggests that TBV does not change in a linear fashion, such that it would become significantly greater or smaller with increasing classifications of obesity [5, 9]. Thus, we cannot say for sure that there is any significant change in TBV in more severe forms of obesity. Second, we note that classifying obesity based on BMI has been critiqued over the past 20 years, and recently alternate forms of obesity classification have been proposed. Our findings may simply reflect the limitations of BMI in predicting the severity of complications in obesity [27, 28, 29].

While the results of this study are compelling, there are some limitations we wish to note. First, our dataset is limited in size, with only 58 data points from obese women and 97 data points from obese men, giving this observational theoretical exercise limited statistical power. Second, the majority (59%) of our data points come from non‐severe obese people, which may impede our ability to compare between these groups and limit our analysis in severe and morbid obesity. However, even in this study, we find differences between men and women, which would benefit from further analysis with a larger female sample size.

The complexity of estimating TBV in obesity, and the simple fact that obesity physiology is understudied, begs the question: should we be estimating TBV in obese people or even normal weight people? Previous comparisons of measured and estimated TBV have not been promising. One study found discrepancies of greater than 500 mL between estimated and measured blood volume in over 60% of cases [26], and a second study found Nadler's formula underestimates blood volume by 15% [19]. While estimating TBV with an algorithm may be convenient, the literature suggests it is less accurate than measurement.

For about 50 years, the gold standard for measuring TBV was NM‐BVA using dual isotope labeling of both RBCs and albumin to separately determine the red cell mass and plasma volume [12]. In years past, this method was cumbersome and time consuming, taking 6–8 h for the examination alone, and thus had little relevance to daily patient care. However, in the early 2000s, several automated benchtop analyzers came on the market, which were an improvement in several ways. First, instead of using labeled red blood cells, these systems utilize I‐131 labeled albumin to calculate TPV. This is then used with the patient's hematocrit to determine TPV, total RCV, and TBV in 90 min or less [12, 13, 30].

Another long‐known technique to measure TBV has also received a recent upgrade. CO re‐breathing exposes a patient to a small, nontoxic amount of CO, which is then absorbed by red blood cells [14, 15]. A sample taken shortly after exposure can determine the TBV by measuring the dilution of CO. Recently, multiple automated benchtop devices were released that utilize this method to measure TBV in no more than 15 min [31, 32].

## 6 Conclusions

In conclusion, we find that the choice of algorithm produces a substantial impact on apheresis procedures, and comparing algorithms with each other in a real‐world dataset produces widely disparate results. Furthermore, none of the formulas we tested to estimate TBV have been validated recently, and the underlying physiology of obesity is poorly studied. It may behoove us as a field to move toward measurement of TBV in obese persons using either NM‐BVA or CO re‐breathing techniques, both of which now have automated analyzers on the market.

## Author Contributions

C.R. devised the study, performed the data analysis and produced figures, and wrote the manuscript. N.S. confirmed results and performed additional statistical analyses and contributed to the manuscript. K.W.‐M. provided the data and provided valuable comments and edits on the manuscript.

## Disclosure

The opinions expressed in this review are those of the authors and do not necessarily represent the views or policies of the National Institutes of Health, the Department of Health and Human Services, or the U.S. Federal Government.

## Ethics Statement

This study was determined by the NIH institutional review board (IRB) to constitute exempt human subjects research per 45CFR 46.104(d) (4)(ii).

## Consent

The authors have nothing to report.

## Conflicts of Interest

The authors declare no conflicts of interest.

## Data Availability

Data is available upon request.
